# HIV Programs for Sex Workers: Lessons and Challenges for Developing and Delivering Programs

**DOI:** 10.1371/journal.pmed.1001808

**Published:** 2015-06-16

**Authors:** David Wilson

**Affiliations:** World Bank, Global HIV/AIDS Program, Washington, D.C., United States of America

## Abstract

There is evidence that HIV prevention programs for sex workers, especially female sex workers, are cost-effective in several contexts, including many western countries, Thailand, India, the Democratic Republic of Congo, Kenya, and Zimbabwe. The evidence that sex worker HIV prevention programs work must not inspire complacency but rather a renewed effort to expand, intensify, and maximize their impact. The PLOS Collection “Focus on Delivery and Scale: Achieving HIV Impact with Sex Workers” highlights major challenges to scaling-up sex worker HIV prevention programs, noting the following: sex worker HIV prevention programs are insufficiently guided by understanding of epidemic transmission dynamics, situation analyses, and programmatic mapping; sex worker HIV and sexually transmitted infection services receive limited domestic financing in many countries; many sex worker HIV prevention programs are inadequately codified to ensure consistency and quality; and many sex worker HIV prevention programs have not evolved adequately to address informal sex workers, male and transgender sex workers, and mobile- and internet-based sex workers. Based on the wider collection of papers, this article presents three major clusters of recommendations: (i) HIV programs focused on sex workers should be prioritized, developed, and implemented based on robust evidence; (ii) national political will and increased funding are needed to increase coverage of effective sex worker HIV prevention programs in low and middle income countries; and (iii) comprehensive, integrated, and rapidly evolving HIV programs are needed to ensure equitable access to health services for individuals involved in all forms of sex work.

## Abstract 2

Reflecting on the "Focus on Delivery and Scale: Achieving HIV Impact with Sex Workers" collection, David Wilson outlines action-oriented recommendations to increase the reach, intensity, and impact of HIV prevention interventions targeted towards sex work.

**Summary Points**

- HIV prevention programs for sex workers, especially female sex workers, are cost-effective.
- There are opportunities to further increase the impact of HIV prevention programs for sex workers and to adapt interventions to a changing context.
- Many sex worker HIV prevention programs are insufficiently guided by understanding of epidemic transmission dynamics, situation analyses, and programmatic mapping; receive limited domestic financing in many countries; are inadequately codified to ensure consistency and quality; and have not evolved adequately to address informal sex workers, male and transgender sex workers, and mobile and internet-based sex workers.
- We recommend increasing our understanding of HIV epidemic transmission dynamics, improving situation analyses and programmatic mapping, increasing domestic financing for sex worker HIV prevention programs where feasible, delivering well-codified, comprehensive programs using “Science of Delivery” principles and developing more effective models to reach informal sex workers, male and transgender sex workers, and mobile and internet-based sex workers.
- Given their marginalization, concerted efforts must be made to ensure sex workers have equitable access to HIV prevention, care, and treatment services, as well as wider health services, particularly for STIs, mental health, and addictions.

## Introduction

The PLOS Collection “Focus on Delivery and Scale: Achieving HIV Impact with Sex Workers” amplifies evidence that HIV prevention programs targeting sex workers, particularly female sex workers, are cost-effective [1–20]. These programs work robustly at scale in a variety of contexts, even with imperfect implementation, and can be implemented with modest resources. In the global north, early female sex worker HIV prevention programs have averted potential HIV epidemics, reduced transmission of other sexually transmitted infections (STIs), and improved population sexual health [21–24]. In Australia, no case of HIV transmission has ever been linked to female sex work independent of drug use [24,25]. In Asia, the early campaign to promote 100% condom use by female sex workers reduced Thailand’s STI and HIV epidemic by up to 90% [26]. In India, community-based targeted interventions reduced India’s epidemic by 60% [27–29]. In challenging urban contexts in Africa, early female sex worker HIV prevention programs have reduced HIV transmission in Matonge in Kinshasa [3], Pumwani in Nairobi, and Bulawayo in Zimbabwe [30], and have subsequently been expanded to national programs in many African countries, including Kenya and Zimbabwe.

The PLOS Collection emphasizes evidence that sex worker HIV prevention programs are robust and cost-effective. However, it must not inspire complacency. Rather, the Collection should inspire renewed determination to expand, intensify, improve the quality of, and maximize the impact of sex worker HIV prevention programs [31]. The Collection aims to promote closer collaboration between practitioners and researchers so that practice is informed by relevant research and research priorities are influenced by the needs of practitioners. The aim is to strengthen research and evaluation with immediate, actionable implications for improved sex worker HIV prevention programs.

The Collection also highlights major challenges in existing sex worker HIV prevention programs, noting the following: sex worker HIV prevention interventions are insufficiently guided by understanding of epidemic transmission dynamics, situation analyses, and programmatic mapping; sex worker HIV and STI services receive limited domestic financing in many countries; many sex worker HIV prevention programs are inadequately codified to increase consistency and quality; and many sex worker HIV prevention programs have not evolved adequately to address informal sex workers, male and transgender sex workers, and mobile- and internet-based sex workers.

These findings underscore the importance of four principles of effective sex worker HIV prevention programs: (i) strengthened programmatic and contextual understanding, especially of the heterogeneity of sex work; (ii) intensified program implementation and delivery of comprehensive, proven interventions; (iii) improved program monitoring and evaluation, including greater use of real-time monitoring for continuous program improvement and constant strategic and tactical adaptations and rigorous program evaluation, to refine understanding of what works, under what circumstances and how; and (iv) predictable, sustained financing for sex worker HIV prevention programs, including increased domestic financing where feasible.

The Collection also delineates the core elements of effective sex worker HIV prevention programs [9,32–35], which are summarized in Table 1 and include: (i) behavior change communication; (ii) condom promotion and distribution; (iii) comprehensive health care, including STI, sexual, and reproductive health care, and mental health and addiction care; harm reduction services; (iv) HIV testing and treatment; (v) solidarity and group empowerment; and (vi) a supportive local and national policy and legal environment.

**Table 1:** Principles of a comprehensive sex worker HIV prevention program.

Pillar	Comprises
Program understanding	Strengthened programmatic and contextual understanding, including the heterogeneity of sex work
Program implementation	Intensified program implementation and delivery of comprehensive, proven interventions
Program delivery	Improved program monitoring and evaluation, including greater use of real-time monitoring for continuous program improvement and constant strategic and tactical adaptations and rigorous program evaluation, to continually refine understanding of what works, under what circumstances, and how
Program financing	Predictable, sustained financing for sex worker HIV prevention programs, including increased domestic financing where feasible

## Recommendations

Based on the gaps identified in the “Focus on Delivery and Scale: Achieving HIV Impact with Sex Workers” Collection and the wider literature, there are discrete clusters of action-orientated recommendations to increase the reach, intensity, and impact of HIV prevention interventions targeted towards sex work (Table 2).

**Table 2:** Components of a comprehensive sex worker HIV prevention program.

Component
• Behavior change communication usually through peer educators
• Condom promotion and distribution
• Comprehensive health care, including STI, sexual, and reproductive health care, and mental health and addiction care
• Comprehensive harm reduction services where there is drug use
• HIV testing and referral to accessible HIV treatment
• Solidarity and group empowerment in sex worker communities
• Fostering a supportive local and national policy and legal environment including reducing violence and harassment, from partners, the community, and the authorities

### HIV Programs Focused on Sex Workers Should Be Prioritized, Developed, and Implemented Based on Robust Evidence

Wherever there is sex work, there is a need to invest in programs to reduce the transmission of STIs as a core public health priority. However, the distribution, scale, and intensity of STI prevention programs targeted towards sex work must be guided by better epidemic analysis, including analysis of HIV transmission dynamics and an understanding of geographic and population heterogeneity. The overall investment in sex worker HIV prevention programs relative to total national spending on HIV prevention should be guided by the fraction of total HIV infections that are due to sex work. In some cases, sound analysis of an HIV epidemic within a country has been undertaken and has led to investment in HIV reduction programs for sex workers. For example, in India a sound epidemic analysis has led to appropriate investments in HIV reduction interventions targeted towards sex work [27,36–38]—India recognized that its epidemic was driven by sex workers in four high burden southern and western states and focused its HIV prevention investments on sex worker HIV prevention programs in these high burden states. In short, the investments followed the epidemic and reflected HIV transmission patterns [38]. In Southern African countries where HIV is highly generalized and hyperendemic, epidemic analysis suggests very different transmission dynamics. For example, behavioral surveys in the highest prevalence countries, such as Swaziland, Botswana, and Lesotho, suggest that a small fraction of men visit sex workers. Even in potential client occupations such as soldiers, police, guards, and truckers, most men report having casual rather than commercial sexual partners [39,40]. In such contexts, studies of sources of new infections and HIV transmission dynamics suggest that sex work contributes only a small fraction of new HIV infections, and investment in HIV programs must reflect these wide epidemic gradations.

Understanding the contribution of sex work to a local HIV epidemic is, however, only part of the solution, as political commitment to address the health needs of sex workers is critical. In some contexts where HIV transmission sources are well characterized and sex work plays a major role, national programs do not prioritize sex worker HIV interventions [35]. Many countries where sex work contributes a significant fraction of new infections allocate a small proportion of their HIV/AIDS budgets to sex worker HIV prevention programs [41].

Situation analyses and programmatic mapping are the foundation for high quality HIV programs. While knowing the HIV prevalence and contribution of sex work to an epidemic at a national level is important for advocacy and broad allocative funding choices, understanding the local context is critical for program management and service delivery. Situation analyses and programmatic mapping are usually the first step in an iterative process that is typically undertaken by effective HIV programs that target HIV in the context of sex work. Effective sex worker HIV prevention programs typically undertake methodical situation analyses to develop typologies of sex work, such as seaters (sex workers who operate from a fixed place) or roamers (sex workers who are mobile), or bar-based, street-based, or home-based, and to identify and map areas where sex workers live and meet clients [42–44]. Programmatic mapping is then used to situate services, such as STI and sexual and reproductive health clinics, assign mobile services, recruit and deploy peer educators, and target outreach and condom distribution. Programmatic mapping also helps to determine and assess coverage, set behavioral and biological targets, and monitor performance. However, in contexts where serious human rights violations occur and sex workers face harassment or arrest from officials or the public, the risks of mapping and enumeration of sex workers outweigh the advantages and discrete programming that does not identify participants as sex workers may be required without systematic mapping and enumeration. Even where human rights are robust, data protection and confidentiality must be carefully maintained, including adherence to best practice guidelines for the protection of geographically coded data that enable authorities to identify the precise location of sex work venues. Such data protection guidelines should be based not only on the current human rights context but prudent assessment of future worst-case changes in human rights [45–47].

The coverage, quality, comprehensiveness, and impact of HIV prevention interventions for sex workers are variable, and consistency may be improved using implementation science [7–9,48–50]. Implementation science is “the study of methods to promote the integration of research findings and evidence into healthcare policy and practice” [51]. The Avahan project shows how implementation science may increase the scope, intensity, and impact of sex worker HIV prevention programs [29,38,52–55]. Avahan used private sector capacity and business models to improve the delivery of a public health service—and specifically to rapidly deliver a large-scale sex worker HIV prevention program in India’s highest burden South Indian states (including Maharastra). Avahan’s approaches included the definition and promotion of a proven approach; an execution focus that put results first by streamlining processes; clarity about HIV prevention targets but flexibility about local adaptations to achieve these targets; strong, decentralized management and mentoring support; clear standard operating procedures and quality enhancement systems; training packages and job aids; the use of real-time data for constant program measurement and strategic and tactical adaptations; and a strong rights and equity focus to ensure that the emphasis on results did not compromise equity. These implementation science lessons may be used to increase the scope, quality, and impact of sex worker HIV prevention and treatment programs.

### Local Political Will and Funding Are Needed to Increase Coverage of Effective Sex Worker HIV Prevention Programs in Low and Middle Income Countries

Many HIV prevention programs focused on sex workers have been internationally financed, including the first sex work programs in the Democratic Republic of the Congo, Kenya, Cameroon, and Zimbabwe [56–58]. Sex worker HIV prevention programs in West Africa were highly reliant on the Canadian International Development Agency regional SIDA-1, 2, and 3 programs [41], and many programs closed when international financing ended. India’s first programs in Tamil Nadu were financed by the United States Agency for International Development in 1992, and subsequent statewide programs were financed by the United Kingdom Department for International Development, the United States Agency for International Development, and later the Bill and Melinda Gates Foundation [59], and the National AIDs Control Programme (NACP)-1, 2, and 3 programs were cofinanced mainly by the Government of India and the World Bank. India has successfully transitioned from international to domestic financing and now largely finances its own HIV program, including sex work interventions. India did so by developing mechanisms for the government to contract civil society partners to implement sex worker HIV prevention programs, developing financial and performance monitoring systems, and by progressively increasing the domestic share of the financing of the national HIV response. [60]. In 2012, the Joint United Nations Programme on HIV/AIDS (UNAIDS) reported that over 90% of all sex worker HIV prevention programs in low and middle income countries relied on international funding [61]. This must be urgently addressed, with domestic financing for sex worker HIV prevention programs where feasible.

### Comprehensive and Integrated Health Programs Are Needed to Ensure Equitable Access to Health Services for Individuals Involved in All Forms of Sex Work

The experience of providing effective health programs for sex workers is also relevant for the wider AIDS debate, without decrying the immense clinical benefits of HIV treatment and the potential for population-level HIV prevention impact, HIV treatment alone is insufficient. Access to treatment requires comprehensive services for sex workers, including actions to overcome stigma and discrimination. Treatment adherence among sex workers has powerful behavioral determinants and is reinforced by sex worker friendly services, peer support, and a supportive policy and legal context. Consistent condom use, reinforced by client programs, offers sex workers additional protection from HIV-infected clients who may or may not be on treatment and virally suppressed. Sex worker-friendly services, including STI and sexual health care, can reduce other STIs, which increase HIV susceptibility. Group solidarity and empowerment creates a powerful impetus for HIV prevention and treatment adherence and is cost-effective [62].

The assertion that sex worker HIV prevention programs are effective applies to formal, self-acknowledged, professional sex workers. However, it is less true for informal non-self-identifying sex work and is even less true for transactional sex (if transactional sex is considered a form of sex work). While transactional sex involves the exchange of gifts, this exchange may be delayed and the link not necessarily acknowledged by either party [63]. For this reason, programmatic and communications approaches to address informal sex work may have more in common with general population interventions than formal sex work interventions. This makes transactional sex harder to address: it is often harder to identify; people who do not self-identify as professional sex workers may be less able to negotiate condom use [64,65]; sexual partners are more willing to use condoms in commercial than casual relationships; it is harder to make condom use normative in casual sex; and it is harder to create group solidarity where there is no discernable group identity.

The HIV response must develop and evaluate intervention models for informal sex work. It must also develop and evaluate better “hotspot” (spatially concentrated nodes of elevated HIV transmission) models to address a gradation of formal and informal sex work and transactional sex. This is urgently needed for several reasons: (i) as HIV epidemics mature and clients perceive formal sex workers and formal sex work venues as “higher risk,” formal networks may disperse and fragment and become harder to define and reach, a process intensified by the increasing role of mobile phones and internet in sexual solicitation [66,67]. These factors may partly account for the decline in “red-light” districts and brothel-based sex work; and (ii) in some countries where women have greater freedom to form sexual partnerships outside marriage, there is a gradation of forms of sex work along a continuum from formal sex work to informal sex work to transactional sex, which may merge into what we consider casual sex or “boyfriend–girlfriend relationships.”

Mobile and internet solicitation is replacing physical venues in some contexts and changing the nature of sex work as it lowers the barriers to entry into sex work and creates larger, more dispersed and fragmented and sometimes more part-time forms of sex work [68–71]. As virtual solicitation replaces physical solicitation, as it is doing rapidly among men who have sex with men, there is a growing danger that programs will continue to focus on the sex work industry of the past and not reach the sex work industry of today and the future. Surveillance, programs, monitoring, and evaluation must rapidly evolve to address mobile- and internet-based sex work or risk irrelevance. This requires developing well-evaluated models that reach sex workers who seek clients through mobile phones or internet, providing them with effective behavior change communication, and linking them to HIV, STI, mental health, and addiction services as needed.

### There Is Need to Increase Programs for Male and Transgender Sex Workers

Male and transgender sex workers are at great risk of stigma, discrimination, gender-based violence, and HIV infection, yet receive fewer programs and services [72–75]. The accelerating global bifurcation of countries who embrace greater rights for sexual minorities, including much of the global north, parts of Latin America, India, Nepal, and South Africa, and countries with restrictive laws and policies (including parts of the Caribbean, the Middle East, the former Soviet Union, and Africa) presents a challenge for intensified male and transgender HIV programs [76,77]. The AIDS movement must continue to advocate for equal rights for sexual minorities, while seeking to expand services for sexual minorities. The global AIDS response must elevate political commitment to address male and transgender sex workers, increase investment in programs for male and transgender sex workers, and evaluate and promote effective models for reaching male and transgender sex workers.

### Concerted Efforts Are Required to Ensure that Sex Workers Receive Equitable Access to Services

Sex workers face a greater burden of HIV and STI infection [78] and a greater risk of mental illness and addiction [79,80]. They also face systematic barriers to accessing appropriate services [81–84]. Sex workers will not achieve equitable access to HIV treatment and health services without concerted strategies to ensure they can overcome these barriers to access and adhere to uninterrupted care. Stigma, discrimination, and criminalization are not only obstacles to HIV treatment access but also to treatment adherence and viral suppression. The fear of arrest and consequent need to hide or move to avoid arrest, together with actual arrest and incarceration without access to services, present major obstacles to HIV treatment initiation and adherence and wider health care and require specially targeted and tailored approaches [84]. In short, extra, concerted effort is needed to overcome the barriers that sex workers face and provide equitable access to services.

## Conclusion

The “Focus on Delivery and Scale: Achieving HIV Impact with Sex Workers” Collection challenges AIDS researchers and practitioners to initiate a new generation of comprehensive sex worker HIV prevention programs for a changing sex work context and provides a framework and tools to do so, while sounding several cautions, particularly concerning informal sex workers, male and transgender sex workers, and mobile- and internet-based sex workers. It underscores the need for concerted effort to ensure marginalized communities of sex workers receive equitable access to HIV and wider health services.

## Abbreviations:

NACP: National AIDs Control Programme
STIs: Sexually transmitted infections
